# Carbohydrate mouth-rinsing does not rescue simulated time trial performance in trained endurance cyclists following a 5-day ketogenic diet

**DOI:** 10.1080/15502783.2025.2598232

**Published:** 2025-12-12

**Authors:** Guy Guppy, James Brouner, Owen Spendiff

**Affiliations:** a Department of Applied Human Sciences, Kingston University London, London, UK

**Keywords:** Ketogenic diet, carbohydrate, mouth-rinse, endurance, cycling, low-carbohydrate

## Abstract

**Background:**

Carbohydrate mouth rinsing (CHO-MR) during periods of fasting or low muscle glycogen availability could provide a more pronounced ergogenic effect compared to fed and high muscle glycogen conditions. However, there is little evidence investigating the efficacy of CHO-MR during periods of low muscle glycogen induced by ketogenic diets. Therefore, this study aimed to investigate the impact of CHO-MR vs. a placebo (PLA-MR) on cycling time trial performance in trained endurance cyclists following their habitual diet (HD) or a 5-day ketogenic diet (KD).

**Methods:**

Eight participants completed baseline testing and four trial conditions. For each trial, participants adhered to either their HD or a KD for 5 consecutive days. During the first 4 days of each dietary phase, they tracked daily nutrition; additionally, they recorded morning fasting blood glucose and *β*-hydroxybutyrate (βHB) levels for the 4 days preceding and the morning of each trial. Each trial comprised a 33.6 km simulated time trial in which rinsing was performed for ten seconds at 7 km intervals.

**Results:**

The 5-day KD significantly increased the time to completion (TTC) compared to HD (*p* < .001). Although no significant differences in TTC were detected between HD + CHO-MR and KD + CHO-MR (*p* = .670), CHO-MR did not restore KD performance to within 2% of HD conditions (±158 s; 4.8%). While a significant main effect for diet on morning fasted blood [βHB] (*p* = .001) was observed on day 5, it was not significantly associated with exercise time (*r*_(14)_ = −.442, *p* = .086). Post-exercise blood [glucose] was significantly higher in the HD + CHO-MR and HD + PLA-MR conditions compared to the KD + CHO-MR (*p* = .038 & *p* = .021), and KD + PLA-MR (*p* = .011 and *p* = .003) conditions, respectively.

**Conclusion:**

The data indicate that repeated 6.4% CHO-MR during endurance cycling is insufficient to overcome performance impairments induced via a 5-day ketogenic diet. This suggests that peripheral substrate availability may constrain the hepatic glucose output in response to central nervous system cues. Further research is required to elucidate how peripheral glycogen stores, central neural drive, and ergogenic interventions interact under low-carbohydrate conditions.

## 1 Introduction

Since the early work of Christensen and Hansen [1] and the introduction of the needle muscle biopsy technique in the 1960s [2,3], carbohydrate (CHO) has been widely accepted as the primary fuel source for optimizing athletic performance [4,5]. However, despite their critical importance, endogenous CHO stores are limited. To overcome this limitation during prolonged exercise, strategies involving the consumption of CHO-rich foods and supplements have been extensively researched and applied in practice [6]. However, high CHO intake combined with CHO malabsorption during exercise is also associated with an increased risk of gastrointestinal distress [7,8]. Consequently, interest in low-CHO, high-fat diets has increased owing to their potential to increase fat oxidation [9], which has been correlated with faster Ironman triathlon times [10]. Moreover, increasing plasma free fatty acid (FFA) availability via a high-fat or ketogenic diet (KD) may also reduce reliance on muscle glycogen [11], which, in turn, reduces the need for high volumes of CHO supplementation during prolonged exercise [9].

Despite the early findings by Phinney and colleagues [12], who showed that endurance performance could be maintained even with muscle glycogen stores reduced by approximately 50% after a four-week KD, evidence has consistently demonstrated an impairment in performance following KDs of less than four weeks in duration [13–16]. However, following a KD of < 4 weeks, impaired performance is primarily linked to reduced muscle glycogen content [17] and the impairment of glycolysis and reduced pyruvate dehydrogenase complex activity [18]. All of these factors lead to earlier and more severe hypoglycemia during prolonged exercise, leading to early termination of exercise [2].

Additionally, early adaptation (<2 weeks) to a KD is often accompanied by symptoms such as fatigue, headaches, and reduced cognitive function [19]. While nutritional ketosis can occur within 2‒4 days, the ability to utilize ketone bodies in place of the diminished glycogen pool may be influenced by training and nutritional status, alongside exercise characteristics [20]. Therefore, organs such as the brain, which derive most of their energy from glucose but have the capacity to utilize ketone bodies as an additional fuel source, may be affected by the lower circulating glucose, impaired glycometabolism and early nutritional ketosis. These physiological and metabolic constraints, particularly those affecting glucose availability and brain metabolism, underscore the need to explore alternative strategies that may support performance under such conditions.

One ergogenic strategy that has gained traction is carbohydrate mouth rinsing (CHO-MR). Early work by Carter et al. [21] highlighted that a temporary exposure of CHO in the oral cavity without ingestion was sufficient to improve endurance performance by approximately 3%. Further work by Chambers et al. [22] established the link between CHO exposure in the oral cavity and activation of the reward and motor control regions of the brain. In recent years, the efficacy of CHO-MR has been shown to be more pronounced in nutritionally compromised states, i.e. fasted [23,24] and low muscle glycogen [25].

Therefore, the present study investigated the effect of repeated CHO-MR on cycling time-trial performance in trained endurance athletes following either a CHO-rich habitual diet (HD) or an isocaloric ketogenic diet (KD) for 5 days. We hypothesized that CHO-MR would enhance performance under both dietary conditions and that it would help restore performance following a KD by mitigating the side effects associated with early ketogenic adaptation. Additionally, we explored whether circulating [βHB] was associated with time-trial performance following each condition. Finally, we investigated whether the power output immediately following each mouth rinse varied across conditions.

## 2 Methods

### 2.1 Participants

Ten well-trained endurance cyclists (*n* = 9 male, *n* = 1 female) volunteered to participate in the present study. Two participants’ data were excluded from the analysis because of not following the prescribed route during the simulated time trials (*n* = 8, *n* = 7 male, *n* = 1 female; mean ± SD; age = 37 ± 6 years, stature = 1.79 ± 9.7 m, baseline body mass = 75.2 ± 12.1 kg, $\dot{V}$ O_2max_ 53.1 ± 9.9 ml/kg/min, resting metabolic rate 9.20 ± 1.26 MJ/day). The inclusion criteria required at least 12 months of endurance training from the study start date, free from any known metabolic diseases, between the ages of 18 and 50, not adhering to either a high-fat (<130 g/day of CHO and >40% of energy intake from fat) or ketogenic diet (<50 g/day of CHO) in the three months preceding the study start date, and blood pressure between 90/60 and 149/90 mmHg. Details of the study aims and procedures were provided to the participants, and written informed consent was obtained before participation. All study procedures were scrutinised and awarded a favourable ethical opinion by the Kingston University London Ethics Committee (no. 3254) and adhered to the principles of the Declaration of Helsinki.

### 2.2 Experimental design

**Figure 1.**
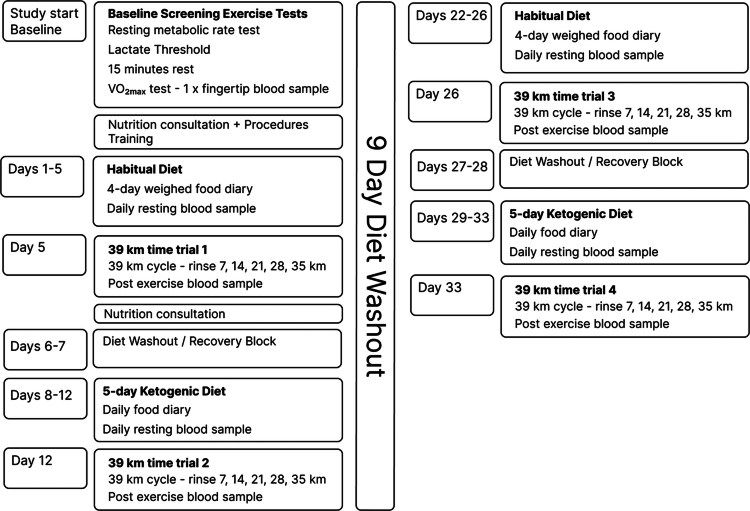
Overview of the study testing procedures and order of the habitual and ketogenic diets.

A crossover study with randomized, double-blind rinsing conditions and a fixed diet sequence was used to investigate the effects of a 5-day KD on endurance cycling performance in combination with CHO-MR and PLA-MR. Participants attended the laboratory on five separate occasions: one for baseline testing (resting metabolic rate, lactate threshold, and $\dot{V}$ O_2max_ tests, followed by a short nutrition counselling session) and four for the 33.6 km simulated time trials. For the 4 days preceding and the day of each simulated time trial, participants were required to adhere to either their habitual diet (HD; trials 1 and 3) or a ketogenic diet (KD; trials 2 and 4), and all food and drink consumed during the first 4 days of each 5-day block were recorded. Furthermore, participants were required to adhere to their normal training schedule throughout the study but were instructed to undertake a rest day in the 24 h preceding each time trial. On the fifth day, participants attended the laboratory in a fed state, compliant with the allocated diet for that experimental trial, to complete body composition assessment and a 33.6 km simulated time trial. In each trial, participants were randomly allocated either 5 × 25  ml CHO-MR or 5 × 25  ml PLA-MR mouth rinses. The ordering of diet conditions was fixed to mitigate any potential residual effects of the KD influencing outcomes during the HD phases. Between the third and fourth visits, an additional 7-day washout period was allocated to further reduce any dietary interference between trials (Figure 1).

### 2.3 Baseline testing

On acceptance to the study, participants were screened for health history, current dietary practices, and blood pressure (M10-IT, Omron Healthcare, Netherlands). Following screening, the participants were assessed for their stature (Harpenden, Holtain Ltd., UK), body mass and composition (MC-980MA Plus BIA, Tanita Europe B.V., Netherlands). Resting capillary blood [glucose] and [lactate] (Biosen C-Line, EKF Diagnostics, Germany) were sampled for confirmation of the absence of metabolic diseases. Following screening and initial assessments, the participants rested for 15 min in preparation for the resting metabolic rate (RMR) test. Upon completion of the rest of the period, the participants were required to lie prone on the plinth, in a darkened, thermoneutral room, wearing a facemask (74500 Series V2, Hans Rudolph Inc., USA) for 20 min while they were assessed via indirect calorimetry (Vyntus CPX, Vyaire Medical GmbH, Germany) [26]. Resting calorie requirements were determined by deducting the first 5 min of data from the RMR test and selecting a 5-min steady-state block from the remaining 15 min, with a coefficient of variation < 10% [26]. $\dot{V}$ O_2_ and $\dot{V}$ CO_2_ gas exchange data were then used to calculate the daily resting calorie targets via the Weir equation [27].

The participants were then fitted with a heart rate chest strap (H10, Polar Electro, Finland), and the cycle ergometer (AtomX, WattBike, UK) was adjusted for ergonomic comfort. The participants then undertook a 10-min self-paced warm-up. Upon completion of the warm-up, the participants were fitted with a facemask connected to the metabolic cart (Vyntus CPX, Vyaire Medical GmbH, Germany). Then, they commenced the lactate threshold test, adapted from Davison et al. [28], which consisted of 3-min stages at an agreed-upon individualised intensity. The starting intensity was assessed based on each participant's perceived fitness and ability. At the end of each 3-min stage, the intensity was increased by 25 W, and the participant provided a fingertip capillary blood sample and a rating of perceived exertion (RPE; 6–20 Borg scale). The test was continually increased by 25 W every 3 min until the blood lactate concentration (BLa) exceeded 4 mmol/L. Inspired and expired gases were recorded throughout on a breath-by-breath basis.

**Figure 2.**
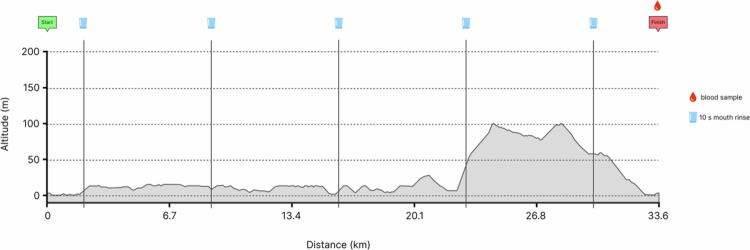
Sugar Cookie course profile for the 33.6 km performance section. Mouth rinses were completed every 7 km, and a fingertip blood sample was taken on cessation of the time trial.

Participants were then given a 15-min rest period before commencing the maximal oxygen uptake test. After the lapsed rest period, participants commenced the maximal oxygen uptake test at a starting intensity equivalent to the participant's lactate threshold. The test was subsequently ramped at 25 W every minute until volitional exhaustion. A final fingertip capillary blood sample was taken 1 min after exercise cessation.

### 2.4 Diet

After the baseline testing session, the participants received nutrition counselling and guidance on the structure and composition of the KD; in addition, they were provided with resources to facilitate adherence and compliance with the KD. The participants were instructed to record all the food and drink consumed in the 4 days preceding each experimental time trial. All diet data were analyzed for macronutrient composition and distribution via SENPRO dietary analysis software (SENPRO, Zereba Software Pty Ltd., UK). During the KD, participants were given the macronutrient goals of <50 g/d CHO after accounting for the fiber content of the foods, >60% fat, with the remaining calories being allocated to protein. A 7-day dietary wash-out period was prescribed to all participants between experimental trials 2 and 3 to ensure that there were no residual effects on subsequent time trials. The participants were not required to report their intake during this time but were instructed to follow their HD. To ensure that body mass remained constant during the study, participants were instructed to consume their resting calorie requirements, as determined from the baseline RMR test, plus calories expended from physical activity each day.

### 2.5 Daily blood analysis

Participants were provided with handheld glucose and ketone meters (FreeStyle Optium Neo, Abbott Diabetes Care, UK) to record one 0.6 μL fasted morning sample of fingertip capillary blood [βHB] and [glucose] for each day of the 4 days preceding and the morning of each trial. The participants were allocated a portable analyser for the duration of the study to reduce the impact of reporting variances between devices. The participants were trained on skin preparation using 70% alcohol swabs, disposable lancets and safe blood collection procedures. Additionally, to ensure compliance in obtaining each daily sample in a fasted state, the participants were instructed to collect the sample immediately upon waking, before consuming any food or drink. Only the Day 5 (pre-trial) values for βHB and glucose were used in the statistical analyses examining associations with time to completion (TTC). The preceding 4 days of data were used to monitor progression into nutritional ketosis but were not included in the inferential statistics. This approach was chosen to reflect the acute metabolic state prior to exercise.

### 2.6 Mouth rinse solutions and protocol

All the mouth rinse solutions were preprepared by the principal investigator and subsequently blinded by allocating a bottle number to each solution by a researcher independent of the study, with the bottle content revealed only to the research team after all the participants had fully completed all the experimental trials. The 1 L premade solutions were kept refrigerated below 5 °C during storage, and 25 ml boluses were extracted from the 1 L solutions on the day of testing and allowed to rise to room temperature before being administered during the experimental trials. Both solutions were indistinguishable in color, texture, odor, and taste. The 1 L premade PLA-MR solution consisted of 150 ml of commercially available artificially sweetened, non-caloric orange concentrate dissolved in 850 ml of water. The 1 L CHO-MR consisted of the same ratio of concentrate to water as the PLA-MR mix, with an additional 64 g of maltodextrin dissolved to form a 6.4% concentration.

### 2.7 Simulated time trials

Prior to the participants completing each time trial, they were assessed for body composition (MC-980MA Plus, Tanita, Japan) to ensure accuracy of in-game performance, and fitted with a chest strap (H10, Polar Electro, Inc., Finland) to assess heart rate. The participants were required to take a 39.2 km route (Sugar Cookie) via a commercially available virtual cycling platform (Zwift Inc., CA, USA), which was linked to an electromagnetically braked cycle ergometer (AtomX, WattBike, UK) and tablet computer (iPad, Apple, USA). The first 5.6 km of the route served as a progressive warm-up, which was not timed. The participants were instructed to complete this section at a self-selected pace reflective of their typical warm-up. Upon reaching the designated start line, the remaining 33.6 km constituted the timed performance segment (Figure 2). The participants were instructed to complete this segment as quickly as possible, with all performance indicators hidden from view. Throughout the full 39.2 km route, participants rinsed a 25 ml solution for 10 s every 7 km, ensuring full oral cavity exposure before expectorating into a container. Upon completion of the route, a 20 µl fingertip capillary blood sample was collected and analysed for blood lactate and glucose (Biosen C-line, EKF Diagnostics, Germany), followed by a 10-min self-paced cooldown.

### 2.8 Statistical analysis

All diet, metabolic, and performance data were analysed using SPSS software (v29.0.2.0, IBM, USA). Normality was assessed using the Shapiro‒Wilk test. All the data are presented as the means ± SD unless otherwise stated. One-way repeated measures ANOVA was employed for analysis of body composition and energy intake. Additionally, two-way repeated measures ANOVA was used to analyze the TTC, power data, blood [glucose], and blood [βHB]. Homogeneity was assessed using Mauchley’s test. Where sphericity was present, a Greenhouse–Geisser correction was used. Additionally, where significant interactions were observed, a post hoc Bonferroni correction was used to reduce the risk of type 1 error. The mean difference (ΔM) is presented with 95% confidence intervals (95% CI) where significant pairwise comparisons exist.

Blood lactate concentrations were not normally distributed; therefore, the main effects of diet and rinsing were analysed using Wilcoxon signed rank test. To test for interactions, a Friedman two-way ANOVA by ranks was used with all pairwise comparisons assessed.

Pearson’s correlations were used to determine whether there was an association between day 5 [βHB] morning fasted blood and exercise time. Spearman’s correlation was used if the data were not normally distributed. Significance was set at *p* < 0.05.

Where no significant differences were found in diet and rinse interactions in TTC, Two One-Sided Tests (TOST) were used to establish if mouth-rinse conditions and diets were practically equivalent. A 2% equivalence margin was set to represent the smallest worthwhile change in well-trained recreational athletes. The sample size was established using an a priori power analysis (G*Power v3.1; [29]). Based on a repeated measures, a within-factors ANOVA design with input values set at an alpha value of 0.05, a beta set to 0.95, an effect size of 0.3, and a correlation among repeated measures of 0.9, a minimum of seven participants were required to detect significant differences.

## 3 Results

### 3.1 Diet and body composition

The macronutrient profile of each respective diet condition is displayed in Table 1. The recorded absolute intake of CHO during the KD was marginally higher (52 ± 13 g/day) than the prescribed value (<50 g/day). As expected, large variances in distribution across all macronutrients were observed between the two diet conditions. In keeping with the isocaloric objectives of the study design, there was no significant main effect of condition on energy intake (F_(2.3, 16.1)_ = .196, *p* = .852; η_p_^2^ = .027). The body composition recorded for each timepoint is displayed in Table 2. No significant main effect on body mass (F_(2.35, 16.42)_ = 2.763, *p* = .086; η_p_^2^ = .283), lean body mass (F_(1.79, 12.53)_ = 2.639, *p* = .114; η_p_^2^ = .122), total body water content (F_(2.3, 16.1)_ = 2.586, *p* = .101; η_p_^2^ = .270) or body fat percentage (F_(2.125, 14.874)_ = .507, *p* = .623; η_p_^2^ = .068) was observed.

**Table 1.** Combined dietary intake during the 4-day weighed food diary period for each diet condition.

Diet	Nutrient	Unit	Mean ± SD
HD	Energy	MJ/day	11.8 ± 2.8
		kJ/kg/d	161 ± 41
	Protein	g/day	118 ± 23
		g/kg/d	1.61 ± 0.3
		%E	17 ± 3
	CHO	g/day	316 ± 84
		g/kg/d	4.32 ± 1.2
		%E	45 ± 5
	Fat	g/day	120 ± 36
		g/kg/d	1.63 ± 0.5
		%E	38 ± 5
KD	Energy	MJ/day	11.4 ± 2.0
		kJ/kg/d	154 ± 29
	Protein	g/day	174 ± 31
		g/kg/d	2.41 ± 0.6
		%E	26 ± 5
	CHO	g/day	52 ± 13
		g/kg/d	0.71 ± 0.2
		%E	8 ± 2
	Fat	g/day	203 ± 47
		g/kg/d	2.77 ± 0.6
		%E	66 ± 5

**Table 2.** Body composition values for baseline and each diet and rinse combination.

Condition	Total body mass (kg)	Lean body mass (kg)	Total body water (%)	Body fat (%)
Baseline	75.2 ± 12.1	62.7 ± 8.2	55.9 ± 5.3	16.1 ± 5.5
HD + CHO-MR	75.1 ± 11.8	61.6 ± 6.9	56.1 ± 4.7	17.3 ± 6.5
HD + PLA-MR	74.5 ± 10.8	61.6 ± 8.1	56.2 ± 4.5	17.0 ± 5.0
KD + CHO-MR	73.5 ± 11.0	60.6 ± 6.5	56.6 ± 5.0	16.9 ± 6.5
KD + PLA-MR	74.2 ± 11.8	61.1 ± 7.4	56.8 ± 5.0	17.0 ± 6.6

### 3.2 Simulated time trials

**Figure 3.**
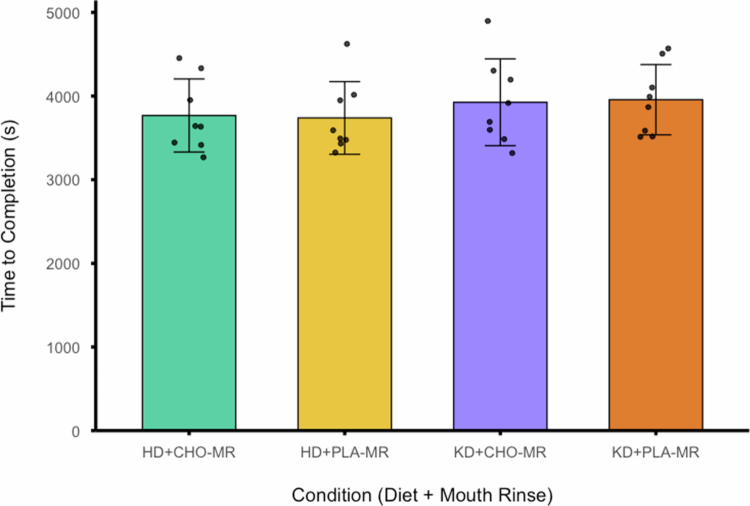
Time to completion of the 33.6 km performance segment of the 39.2 km simulated time trial for each condition.

A significant main effect for diet was observed (F_(1, 7)_ = 54.482, *p* < .001; η_p_^2^ = .886). After a 5-day KD, the TTC was 4.8% slower (3941 ± 456 s) compared to the participant’s HD was (3753 ± 421 s). There was no significant difference in the TTC between mouth-rinse conditions, independent of diet (F_(1, 7)_ = .001, *p* = .974; η_p_^2^ = .000), and no difference in diet and rinse interaction was observed (F_(1, 7)_ = .198, *p* = .670; η_p_^2^ = .027) (Figure 3). Although no significant difference was detected between the CHO-MR conditions of either diet, TOST revealed that a mean difference of 158 s exceeded the 2% equivalence margin (±75 s).

### 3.3 Power output

**Figure 4.**
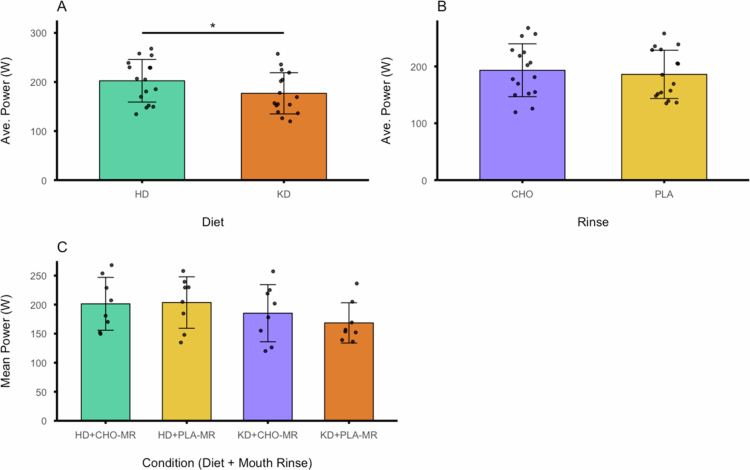
(A) Mean power output across the 33.6 km performance section of the 39.2 km simulated time trial for each diet condition. (B) Mean power output for each rinse condition. (C) Mean power output for each diet and rinse combination.

The average power output was significantly greater (*F*_(1, 7)_ = 70.832, *p* < .001; η_p_^2^ = .910) when adhering to the HD compared to the KD (Figure 4a). However, no significant difference (*F*_(1, 7)_ = 1.317, *p* = .289; η_p_^2^ = .158) was observed between CHO-MR and PLA-MR (Figure 4b). Similarly, no significant difference (*F*_(1, 7)_ = .814, *p* = .397; η_p_^2^ = .104) was detected in the diet and rinse interactions (Figure 4c).

**Figure 5.**
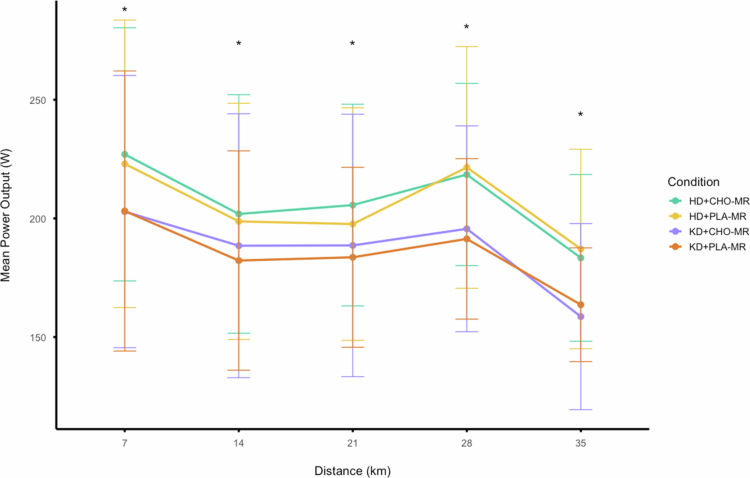
Mean power output for the 500 m immediately following each prescribed mouth rinse during the 33.6 km performance segment. * denotes a significant (*p* < 0.05) main effect between diet conditions within each 7 km segment.

Figure 5 displays average power profiles for each diet and rinse condition, measured across the 500 m distance immediately following each mouth rinse during the simulated time trial. Analysis of the average power output for each 500 m segment immediately following each mouth rinse revealed significant differences between HD and KD for 7km (*F*_(1, 7)_ = 18.744, *p* = .003; η_p_^2^ = .728), 14 km (*F*_(1, 7)_ = 14.092, *p* = .007; η_p_^2^ = .668), 21 km (*F*_(1, 7)_ = 11.475, *p* = .012; η_p_^2^ = .621), 28 km (*F*_(1, 7)_ = 26.890, *p* = .001; η_p_^2^ = .793) and 35 km (*F*_(1, 7)_ = 23.383, *p* = .002; η_p_^2^ = .770), respectively. Additionally, no significant difference was observed between CHO-MR and PLA-MR for 7km (*F*_(1, 7)_ = .163, *p* = .698; η_p_^2^ = .023), 14 km (*F*_(1, 7)_ = 1.552, *p* = .253; η_p_^2^ = .181), 21 km (*F*_(1, 7)_ = 1.477, *p* = .264; η_p_^2^ = .174), 28 km (*F*_(1, 7)_ = .020, *p* = .892; η_p_^2^ = .003) and 35 km (*F*_(1, 7)_ = .531, *p* = .490; η_p_^2^ = .070), respectively. Furthermore, no significant interaction was observed between diet and rinse conditions for 7 km (*F*_(1, 7)_ = .069, *p* = .801; η_p_^2^ = .010), 14 km (*F*_(1, 7)_ = .041, *p* = .845; η_p_^2^ = .006), 21 km (*F*_(1, 7)_ = .043, *p* = .842; η_p_^2^ = .006), 28 km (*F*_(1, 7)_ = .456, *p* = .521; η_p_^2^ = .061) and 35 km (*F*_(1, 7)_ = .014, *p* = .910; η_p_^2^ = .002), respectively.

### 3.4 Blood metabolites

**Figure 6.**
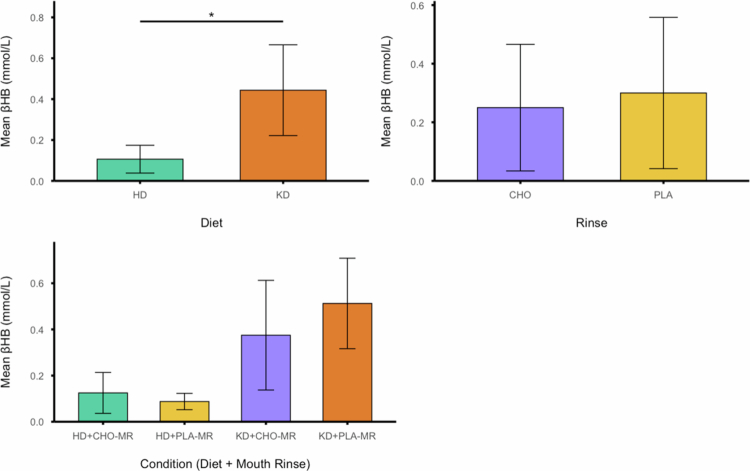
(A) Day 5 morning fasted blood [βHB] for each diet condition. (B) Day 5 morning fasted blood [βHB] for each rinse condition. (C) Day 5 morning fasted blood [βHB] for each diet and rinse combination.

**Figure 7.**
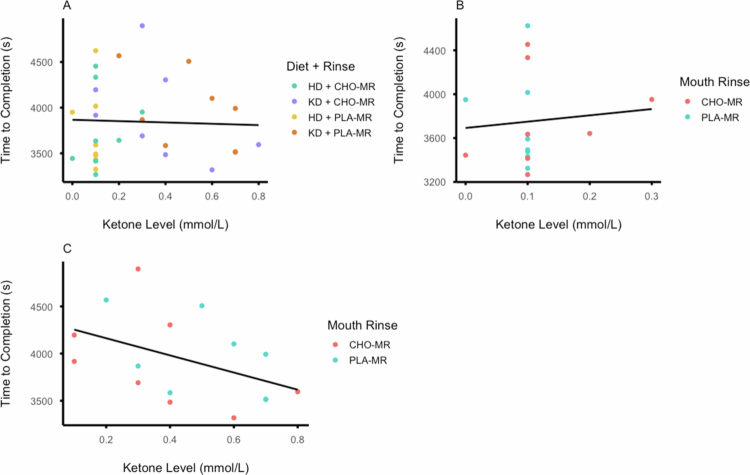
(A) Day 5 morning fasted blood [βHB] and time to completion with all conditions combined. (B) Day 5 morning fasted blood [βHB] and time to completion for the habitual diet. (C) Day 5 morning fasted blood [βHB] and time to completion for the ketogenic diet.

Day 5 morning, fasted blood [βHB] for each diet and rinse condition is presented in Figure 6. A significant main effect of diet on day 5 morning fasted blood (βHB) was detected (F_(1, 7)_ = 26.169; *p* = .001; η_p_^2^ = .789); however, no significant main effect was detected for rinsing (F_(1, 7)_ = 3.111; *p* = .121; η_p_^2^ = .308), and no significant interaction was detected between diet and rinsing (F_(1, 7)_ = 3.465; *p* = .105; η_p_^2^ = .331). Additionally, three separate correlation analyses were conducted to examine the relationship between day 5 morning fasted blood [βHB] and TTC: (1) across all trials regardless of diet or mouth rinse condition (Figure 7a), (2) within the HD condition only (Figure 7b), and (3) within the KD only (Figure 7c). There was no significant correlation between day 5 morning fasted blood [βHB] and TTC for all trials combined (ρ_(32)_ = .092, *p* = .613) or within the HD only (ρ_(16)_ = .166, *p* = .538) or within the KD only (r_(16)_ = −.442, *p* = .086).

**Figure 8.**
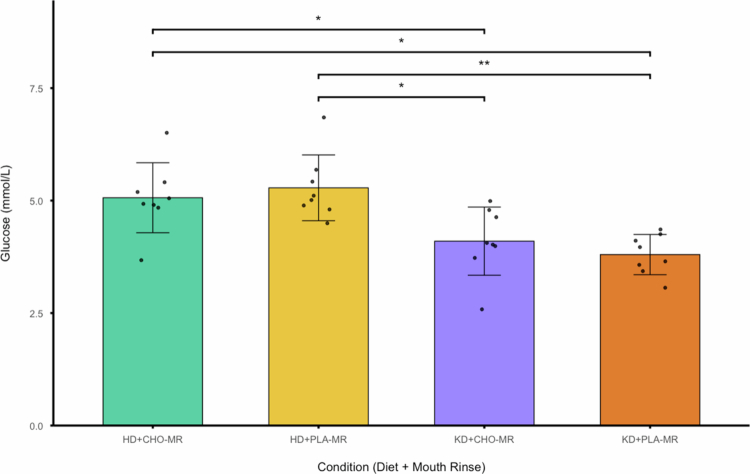
Post-exercise [glucose] across diet × rinse conditions. * denotes *p* < 0.05, ** denotes *p* < 0.01.

Post-exercise blood [glucose] was analysed using a two-way repeated measures ANOVA (Figure 8). A significant main effect of diet was observed (F_(1, 7)_ = 12.573; *p* = .009; η_p_^2^ = .642; HD = 5.17 ± .74 mmol/L vs. KD = 3.95 ± .62 mmol/L), however, no main effects were observed for rinse (F_(1, 7)_ = .057; *p* = .819; η_p_^2^ = .008; CHO-MR = 4.58 ± .89 mmol/L vs. PLA-MR = 4.54 ± .96 mmol/L). A significant interaction effect between diet and rinsing on blood [glucose] was also observed (F_(1, 7)_ = 11.012; *p* = .013; η_p_^2^ = .611). Post hoc pairwise comparisons revealed that post-exercise blood [glucose] were significantly higher in the HD + CHO-MR condition compared to KD + CHO-MR (ΔM = .97; 95% CI [.07 1.86]; *p* = .038) and KD + PLA-MR conditions (ΔM = 1.26; 95% CI [.40 2.13]; *p* = .011). Similarly, post-exercise blood [glucose] were significantly higher in the HD + PLA-MR condition compared to KD + PLA-MR (ΔM = 1.48; 95% CI [.71 2.26]; *p* = .003) and KD + CHO-MR (ΔM = 1.19; 95% CI [.24 2.13]; *p* = .021).

Analysis of post-exercise [BLa] revealed a significant main effect for diet (Z = −2.12; *p* = .034; HD = 4.08 ± 2.39 mmol/L vs. KD = 3.14 ± 1.68 mmol/L) and a non-significant main effect for rinse (Z = −.362; *p* = .717; CHO-MR = 3.71 ± 2.34 mmol/L vs. PLA-MR = 3.51 ± 1.88 mmol/L). Additionally, a significant interaction effect between diet and rinsing on [BLa] was detected (X^2^_(3)_ = 12.30; *p* = .006). Pairwise comparisons revealed a significant difference between KD + PLA-MR and HD + PLA-MR (ΔM = 1.56; Z = 3.49; *p =* .003), and all the other comparisons did not reach statistical significance (*p* > .05).

## 4 Discussion

The present study examined the effect of repeated CHO-MR on simulated cycling time-trial performance in trained endurance cyclists following either a CHO-rich HD or an isocaloric 5-day KD. To the authors’ knowledge, this is the first study to investigate the impact of the CHO-MR during a 5-day KD. It was hypothesized that CHO-MR would enhance cycling performance and restore it to the levels observed with a CHO-rich diet when used after a 5-day KD compared to a PLA-MR under the same conditions. Our results revealed several key findings:

(1) Performance was reduced by 4.8% following a 5-day KD compared to a CHO-rich HD, regardless of CHO-MR;
(2) CHO-MR did not influence time to completion compared to PLA-MR under either diet condition;
(3) Repeated 6.4% CHO-MR after a 5-day KD did not restore performance to the level observed with CHO-MR combined with a CHO-rich HD;
(4) Although the correlation observed between day 5 morning, fasted blood [βHB] and TTC was not significant (*r*_(16)_ = –.442, *p* = .086), the magnitude of the correlation reflects a moderate to large effect size [30], suggesting a possible association that may warrant further investigation.

Adherence to a KD initiates a metabolic shift in substrate use towards fat and ketone bodies, which is compounded by a marked reduction in whole-body glycogen content. Seminal work by Bergström et al. [2] demonstrated that, following glycogen-depleting exercise, muscle glycogen resynthesis remained 50% lower after only 3 days of a high-fat diet. Consequently, metabolically active tissues, particularly the brain, which relies heavily on glucose [31], may experience impaired function when glucose availability is restricted. Whilst the time frame for ketogenic adaptation is heavily disputed both in the lay and scientific literature, with suggestions that increased fat oxidation could occur within 3–7 days and that enzymatic activity stabilizing within 2–3 weeks [32], the negative impact of short-term carbohydrate restriction on exercise performance is well documented [1–3,13–16]. Our observed 4.8% reduction in performance following a 5-day KD aligns with this literature and reinforces the notion that the metabolic shift and change in endogenous glycogen content that takes place in the early stages of ketogenic adaptation (<2 weeks) is sufficient to impair performance [14].

While the performance decrease after following a 5-day KD, as observed in the present study, was expected, the lack of performance restoration when CHO-MR was used under these conditions was unexpected. CHO-MR has been shown to activate oral receptors that stimulate brain regions associated with reward and motor control, such as the anterior cingulate cortex and striatum [22]. This central nervous system stimulation is thought to increase motivation and reduce perceived exertion, particularly under conditions of low carbohydrate availability, where the ergogenic effect of CHO-MR is more pronounced [23–25]. Given that short-term KD markedly reduces muscle glycogen and perturbations in glucose homeostasis, it is common for individuals to experience side effects such as fatigue, dizziness, and decreased energy [19]. Consequently, it was hypothesized that the CHO-MR might offset some of these central limitations. However, our findings show that while CHO-MR reduced the difference in performance between diet conditions to 3.9%, it was still not sufficient to reach the 2% equivalence margin. Therefore, the central effects of a 6.4% CHO-MR solution may be insufficient at overcoming the disruption caused by the shift in peripheral substrate availability imposed by acute CHO restriction via a KD.

One such observation when carbohydrate intake is restricted is the reduction in hepatic glycogen stores, with an overnight fast of ~15 h being sufficient to reduce the liver glycogen content by 34% in healthy control subjects [33]. By reducing the hepatic glycogen content prior to exercise, the ability for the blood [glucose] to be maintained is compromised, potentially leading to exercise-induced hypoglycemia (EIH), which has been implicated in impaired performance during prolonged exercise. In our current study, we observed blood [glucose] of 4.1 and 3.8  mmol/L immediately post-exercise in the KD + CHO-MR and KD + PLA-MR conditions, respectively. In contrast, the blood [glucose] in the HD + CHO-MR and HD + PLA-MR conditions were 5.1 and 5.3  mmol/L, respectively. These findings suggest that the ergogenic effects of CHO-MR may be contingent on adequate peripheral substrate availability and that CHO-MR may attenuate the decline in blood [glucose]. While CHO-MR may activate central pathways involved in motivation and motor control, its efficacy appears limited when perturbations in glucose homeostasis are present.

Although this study is, to our knowledge, the first to examine the efficacy of CHO-MR during nutritional ketosis, previous research has demonstrated improvements in fasted [23] or low muscle glycogen states [34]. Lane et al. [23] found that blood [glucose] was maintained across both CHO-MR and PLA-MR conditions following a 12 h fast. In contrast, our findings revealed a significant reduction in post-exercise blood [glucose] under both KD conditions, which may be attributed to the extended duration of carbohydrate restriction and consequential reduction in glycogen. These results suggest that the ergogenic potential of CHO-MR may be constrained by peripheral substrate availability. Given that Lane’s participants maintained sufficient circulating glucose even in the placebo condition, it is plausible that CHO-MR’s impact on performance depends on an interaction between central neural activation and the availability of metabolic substrates.

Recent evidence further supports our findings that blood [glucose] may be a critical factor in the ergogenic potential of CHO-MR when adhering to a KD. Carpenter et al. [35] demonstrated that in chronically keto-adapted athletes, CHO ingestion immediately prior to exercise significantly improved endurance performance, whereas multi-day CHO loading had no effect. These findings suggest that the timing of carbohydrate intake may be crucial for maintaining euglycemia and supporting the central drive during exercise. Similarly, Prins et al. [9] showed that minimal CHO supplementation (10 g/h) during exercise was sufficient to eliminate exercise-induced hypoglycemia and improve time-to-exhaustion performance by 22%, regardless of whether athletes were following a high- or low-CHO diet. These findings imply that the maintenance of blood [glucose], rather than muscle glycogen per se, may be the limiting factor during prolonged exercise under low-CHO conditions. Furthermore, these differences in study designs compared to our design suggest that the ingestion of CHO prior to or during exercise is critical to mitigate impairments in performance when the glycogen pool is depleted.

Moreover, the participants in our study followed a KD for only 5 days, representing a phase of early adaptation, whereas Carpenter et al. and Prins et al. utilized designs where the participants had undergone weeks to months of ketogenic adaptation. Long-term adaptation to a KD has been shown to normalize blood [glucose] and maintain muscle glycogen content consistent with a high-CHO diet [36]. Therefore, the significant reduction in post-exercise blood [glucose] observed in our study during KD conditions may reflect incomplete metabolic adaptation, reduced glycogen stores and impaired glucose mobilization. These factors likely contributed to the lack of CHO-MR efficacy, suggesting that both substrate availability and metabolic adaptation are essential for CHO-MR to exert its ergogenic effects under nutritional ketosis.

## 5 Limitations

One limitation of the present study was the free-living conditions of the diet; while participants were educated and provided resources on the structure and composition of the KD, there was considerable variability in the distribution of fat, carbohydrate and protein intake. Some participants overcompensated with protein ingestion in place of carbohydrates, which may have hindered their performance due to the limited role of protein in energy pathways while in a fed state [37]. Additionally, although every attempt was made to blind the participants to the distance and time during the time trials, because of the set points used to deliver the mouth rinse at equal intervals, participants may have made a mental note that corresponded to visual cues on the screen, therefore, possibly deploying pacing strategies across time trials depending on how they were feeling. Finally, the present study did not use a no-rinse control, as proposed by Gam et al. [38]; therefore, we were unable to establish whether the presence of a liquid in the oral cavity affected cycling performance following a short-term ketogenic diet.

## 6 Conclusion

In summary, repeated CHO-MR did not restore cycling time-trial performance after a 5-day KD. While CHO-MR is an effective ergogenic aid during exercise in low muscle glycogen conditions [23,34], its effectiveness may be insufficient to counteract the metabolic adaptations caused by CHO restriction induced by a KD. Furthermore, the efficacy of CHO-MR may be predicated on the fine balance between central neural activation and downstream substrate availability to act upon these stimuli. Future research should examine the effectiveness of CHO-MR during longer-term ketogenic diets and investigate the effects of different CHO mouth rinse concentrations under conditions of sustained nutritional ketosis.

## Data Availability

Data are available from the corresponding author upon reasonable request.
